# Glucocorticoids improve severe or critical COVID-19 by activating ACE2 and reducing IL-6 levels

**DOI:** 10.7150/ijbs.47652

**Published:** 2020-06-27

**Authors:** Zhen Xiang, Jialin Liu, Dake Shi, Wei Chen, Jun Li, Ranlin Yan, Yufang Bi, Weiguo Hu, Zhenggang Zhu, Yingyan Yu, Zhitao Yang

**Affiliations:** 1 Department of General Surgery of Ruijin Hospital, Shanghai Institute of Digestive Surgery, and Shanghai Key Laboratory for Gastric Neoplasms, Shanghai Jiao Tong University School of Medicine, 200025, Shanghai, China.; 2 Department of Critical Care Medicine, Ruijin Hospital, Shanghai Jiaotong University School of Medicine, 200025, Shanghai, China.; 3 Department of Infection Control, Ruijin Hospital, Shanghai Jiaotong University School of Medicine, 200025, Shanghai, China.; 4 Department of Pulmonary and Critical Care Medicine, Ruijin Hospital, Shanghai Jiaotong University School of Medicine, 200025, Shanghai, China.; 5 Department of Endocrinology and Metabolism Disease, Ruijin Hospital, Shanghai Jiaotong University School of Medicine, 200025, Shanghai, China.; 6 Emergency Department, Ruijin Hospital, Shanghai Jiaotong University School of Medicine, 200025, Shanghai, China.

**Keywords:** COVID-19, SARS-CoV-2, ACE2, Glucocorticoids, Drug repurposing

## Abstract

COVID-19 is a public health emergency that has rapidly spread to over 200 countries and regions, and no effective treatment has been established to date. Severe and critical cases have been associated with higher mortality due to acute respiratory distress syndrome (ARDS) and cytokine storm. Based on the novelty and recent emergence of COVID-19, no effective treatment regimen has been identified, thus prompting clinicians to engage in drug repurposing to address the immediate therapeutic need. This study focused on the molecular target angiotensin-converting enzyme 2 (ACE2) of SARS-CoV-2 and screened a group of ACE2 agonists by bioinformatics. Glucocorticoids are a type of ACE2 activator. We verified the efficacy of nine chemicals on regulating ACE2 expression in human GES-1, an upper digestive tract epithelial cell line, and THP-1, a human monocyte cell line, and found that several glucocorticoids imparted activating effects on ACE2 in both cell lines. The drugs triciribine and kinetin riboside activate ACE2 expression or inhibit IL-6 production in macrophages to some extent. In addition, we compared the efficacies of several glucocorticoids. Hydrocortisone showed the strongest effect on ACE2 activation, followed by prednisolone, dexamethasone, and methylprednisolone. We retrospectively analyzed the therapeutic efficacy of nine severe or critical patients from a cohort of 90 COVID-19 cases, who received medium to small doses of glucocorticoids from our integrated medical team in Wuhan. Seven out of nine patients revealed significant improvement in clinical parameters and chest CT images. This study provides experimental and clinical evidence that medium-to-low-dose glucocorticoids may play a protective role in the respiratory and digestive systems by activating ACE2 and suppressing cytokine storm.

## Introduction

From the end of 2019 to the beginning of 2020, an acute respiratory disease known as COVID-19 and caused by SARS-CoV-2 had spread throughout China and the rest of the world. COVID-19, combined with severe acute respiratory syndrome (SARS) in 2002 and Middle East respiratory syndrome (MERS) in 2012, are three zoonotic infectious diseases caused by β-coronaviruses that occurred in the last 20 years 1-5. Since β-coronaviruses are not common pathogens to humans, the general public essentially lacks natural immunity to SARS-CoV-2. Thus, this sudden global pandemic has currently no approved specific antiviral agents, and the development of therapeutic drugs is imperative.

Similar to SARS-CoV, SARS-CoV-2 uses the receptor, angiotensin-converting enzyme 2 (ACE2), to infect human cells. The virus spike protein (S protein) would bind to the ACE2 of cells, and largely depleting host cells of this specific receptor 6,7. Some researchers have targeted ACE2, blocking the viral S protein from binding to it. ACE2 is a member of the angiotensin-converting enzyme (ACE) family. It was first cloned from 5' sequencing of a human heart failure ventricle cDNA library in 2000. ACE2 shares homology with ACE in terms of their catalytic domain and provides different key functions in renin-angiotensin-aldosterone system (RAAS). ACE cleaves angiotensin I to generate angiotensin II, whereas ACE2 reduces angiotensin II levels. Thus, ACE2 plays a protective role in the respiratory system, cardiovascular system, and kidneys 8-10. However, SARS-CoV-2 infection induces a reduction in ACE2 levels and disrupts its protective function in the respiratory and cardiovascular systems, as well as other important organs. Patients diagnosed with COVID-19 rapidly deteriorate to severe or critical conditions 11,12. Therefore, taking inhibitory drugs against SARS-CoV-2 or activating ACE2 may be potential therapeutic strategies for treatment of this infectious disease. Thus, there is an immediate need to identify drugs that could improve multi-organ deterioration in severe or critical COVID-19 patients.

Drug development involves two major strategies. The first strategy consists of screening new drugs from the beginning; however, this is time-consuming and often laborious. The second strategy is drug repurposing, also known as drug repositioning (i.e., older drugs, new uses), which refers to screening drugs with potential intervention of the target. Repurposed drugs requires the approval of the FDA for clinical use or use in clinical trials in order to change its clinical utility 13,14. The latter strategy has the advantages of being fast, safe, and inexpensive. Because old drugs are already on the market, this procedure can save a lot of experiments on drug toxicology, pharmacokinetics, and other pre-experiments. Repurposed drugs are generally safe and have no associated adverse side effects. Drug repurposing research is a big data-driven task that highly relies on bioinformatics and chemical informatics 15,16. Researchers use computer programs to analyze gene profiles, and then match the small compounds in the database. The candidate compounds are outputted with marked scores. Compounds with higher scores are considered highly reliable. Then, scientists and physicians verify the therapeutic efficacies of candidate compounds by subsequent experiments or clinical practice.

In this study, we used drug repositioning to screen a group of ACE2 agonists (20 compounds) through cross-database analysis. We verified the activating effect of nine available compounds for ACE2 on human epithelial cells and monocytes. Glucocorticoids are candidate agonists of ACE2. We further analyzed our glucocorticoid therapeutic experience involving nine cases of severe and critical COVID-19 from a cohort of 90 patients in Wuhan who were hospitalized by the medical rescue team of Shanghai Ruijin Hospital. Clinically, the safety and effectiveness of glucocorticoids on improving severe and critical COVID-19 have been confirmed.

## Results

### The basic expression of ACE2 in 1,019 human tumor cell lines from 26 tissue types

**Figure 1:**
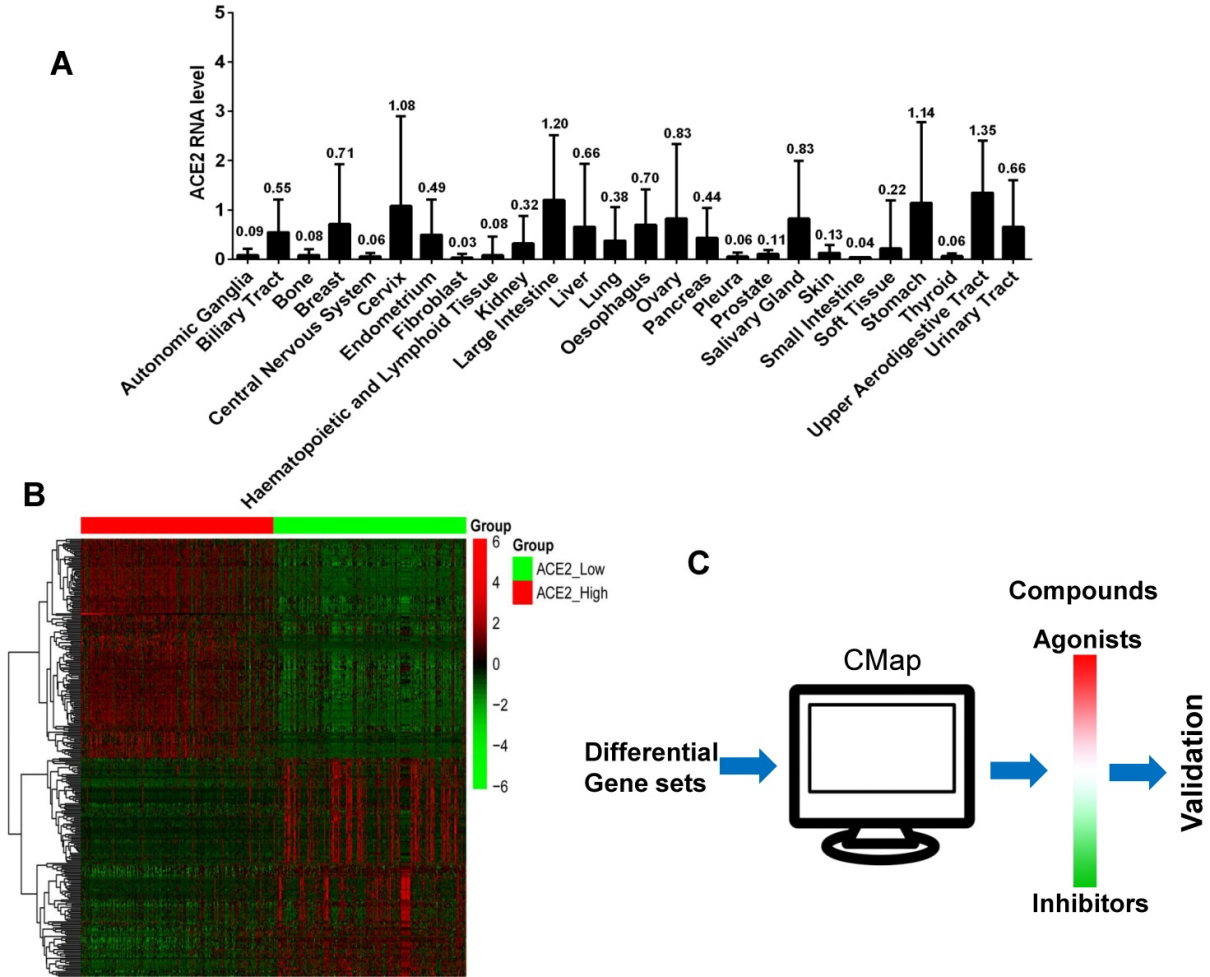
** ACE2 expression analysis and candidate agonists screen. A).** The mean values of ACE2 gene expression of 1,019 tumor cell lines that originated from 26 types of human tissues. The horizontal axis represents cell line origin, and the vertical axis indicates the mRNA expression level of the ACE2 gene. **B).** The heat map shows the mRNA expression values (including the ACE2 gene) of 150 highly elevated or significantly decreased differential genes between the ACE2^high^ and ACE2^low^ groups (fold-change ≥ 1.5, P < 0.01). The red color represents increased mRNA expression levels, and green indicates decreased mRNA expression levels. **C).** The schematic of agonist screening for ACE2 in the CMap database.

We analyzed the ACE2 expression of 1,019 tumor cell lines that originated from 26 types of tissues in the CCLE database. The expression levels and their tissue origin are presented in Figure 1A. Tumor cells from the upper respiratory and digestive tract epithelium exhibited the highest ACE2 expression levels, followed by cell lines of the large intestine, stomach, cervix, and salivary gland. The remaining cell lines from other tissues showed different ACE2 expression levels.

### Screening ACE2 agonists by differentially expressed genes between ACE2^high^ and ACE2^low^

Based on mRNA expression levels of ACE2 in 1,019 cell lines, we divided the ACE2 values into high (n=340), medium (n=339), and low (n=340) groups. Three genetic data sets were obtained. We compared the differentially expressed genes between the ACE2^high^ and ACE2^low^ groups, extracted the top 150 significantly elevated genes (fold-change ≥ 1.5, P < 0.01, Figure 1B), and used these as inputs of the CMap database. The computer matched the compounds that may cause the above differential gene profiles (Figure 1C). The resulting compounds that matched the ACE2^high^ group were considered as ACE2 agonists. Table 1 shows the CMap scores, which represent the matching scores for each compound using 100 full marks, and the closer to 100, the more accurate the match.

### ACE2 expression of human epithelial cells could be activated by glucocorticoids or other agonists

**Figure 2:**
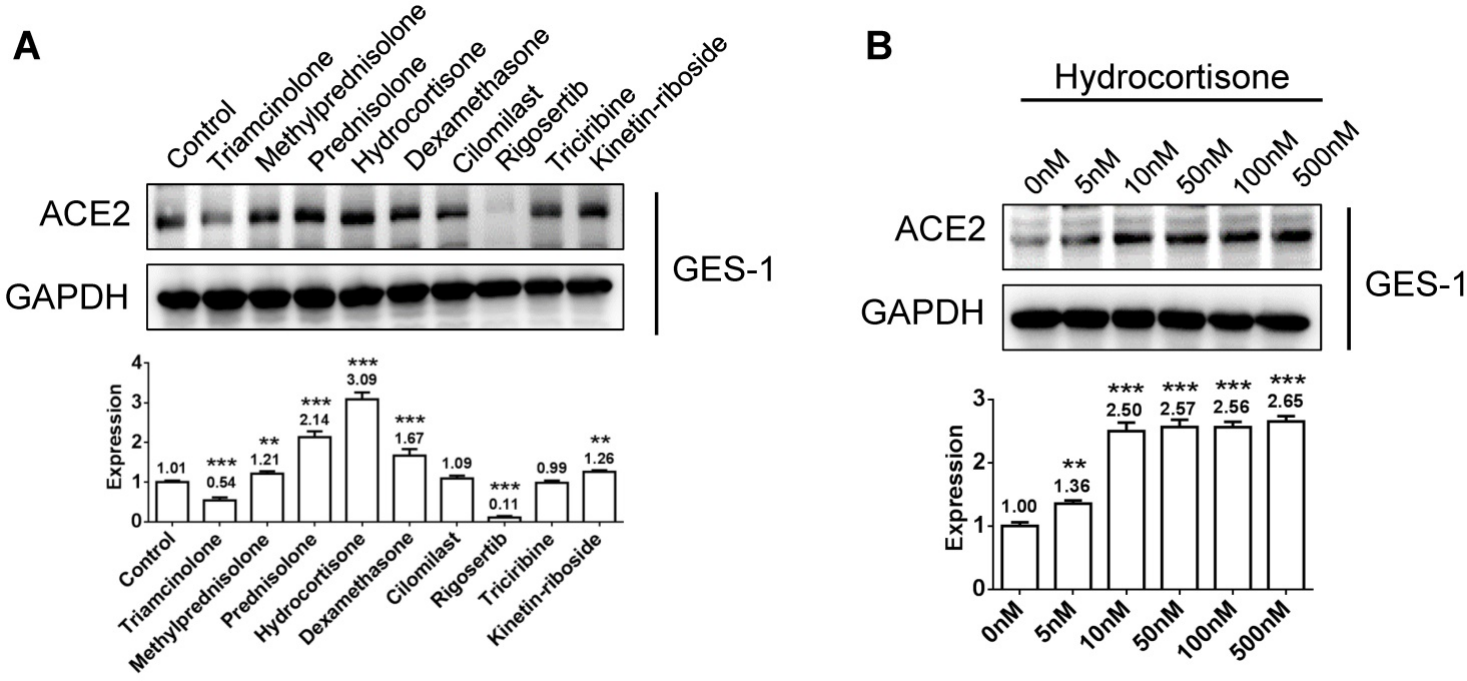
** Activating effects of nine chemicals on ACE2 expression in the human gastric epithelial cell GES-1.** A) Hydrocortisone revealed the strongest activating effect on ACE2 expression, followed by prednisolone, dexamethasone, and methylprednisolone. The candidate chemical kinetin riboside also exhibited an activating effect on ACE2 expression. B) ACE2 expression induced by hydrocortisone is concentration dependent, although a low dose of 10 to 100 nm is sufficient to activate ACE2 expression. “*” represents comparison with the “control” or “0 nm” groups. *P < 0.05, **P < 0.01, ***P < 0.001.

Because the epithelial cells of the respiratory and digestive tracts are susceptible targets of SARS-CoV-2, we verified the regulatory effects of several candidate agonists of ACE2 expression on available normal human epithelial cells. GES-1, a normal digestive epithelial cell line, has been maintained in our laboratory. Five commercially available agonists utilized in pandemics, including triciribine, rigosertib, kinetin-riboside, triamcinolone, and cilomilast were assessed. The functions of four commonly used glucocorticoids such as hydrocortisone, prednisolone, methylprednisolone, and dexamethasone were compared. After treatment of GES-1 cells with 100 nm glucocorticoids or chemicals for 48 h, we examined the protein expression levels of ACE2. Compared to the blank control, hydrocortisone revealed the strongest activating effect on ACE2 expression, followed by prednisolone, dexamethasone, and methylprednisolone. Candidate chemical kinetin riboside also exhibited an activating effect on ACE2 expression (Figure 2A). We further examined the effective doses of hydrocortisone from 0 to 500 nm and found that the activating effect on ACE2 expression is concentration-dependent, but low doses ranging from 10 to 100 nm are sufficient in activating ACE2 expression (Figure 2B).

### Glucocorticoids impart stronger effects on suppressing interleukin-6 release and activating ACE2 expression in macrophages compared to other chemicals

**Figure 3:**
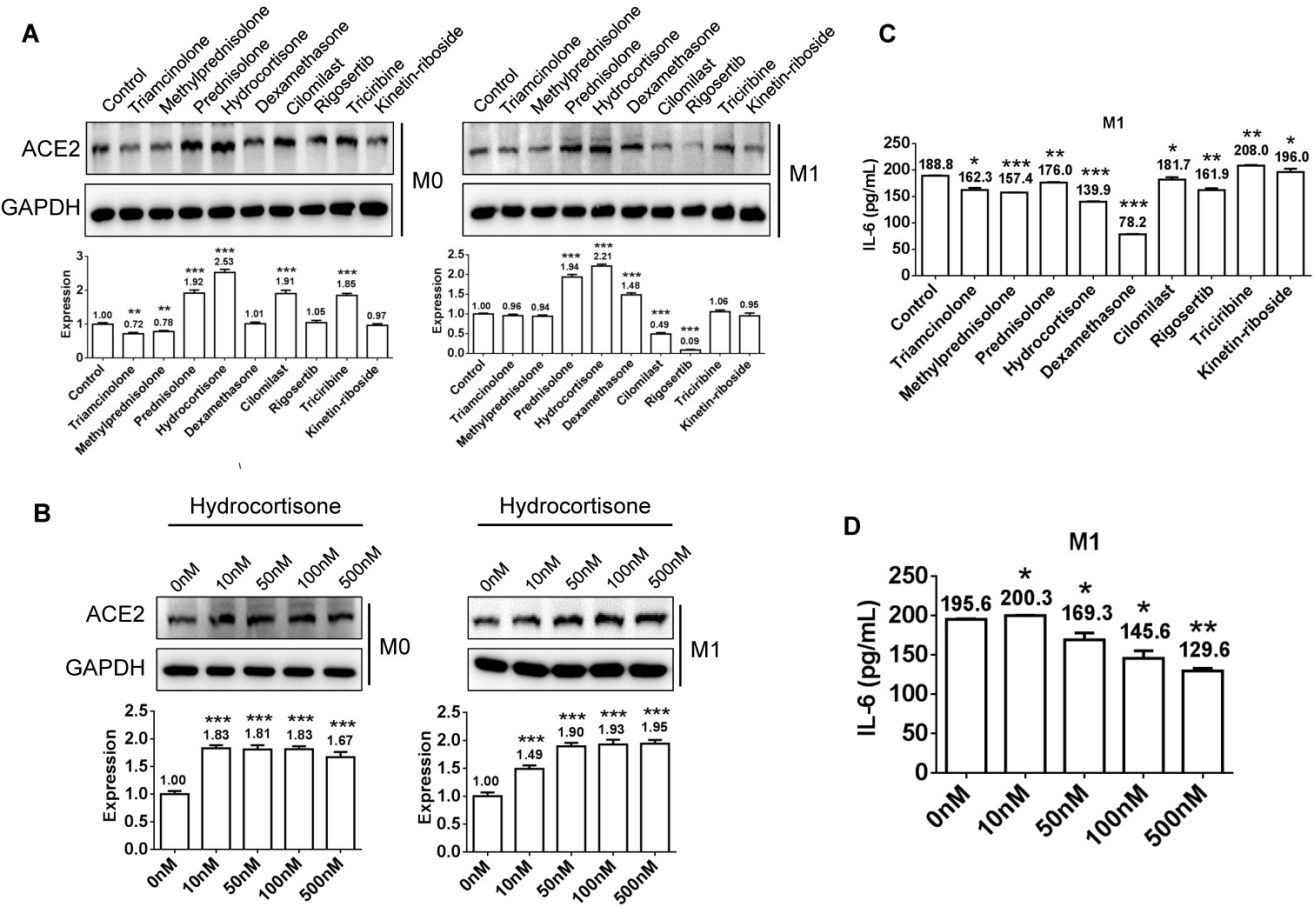
** Activation of ACE2 expression and inhibition of IL-6 secretion in macrophages using glucocorticoids or other chemicals. A**) Several glucocorticoids and candidate agonists activated ACE2 expression in M0 macrophages, whereas only glucocorticoids imparted an activating effect on M1 macrophages. **B)** Low-dose hydrocortisone within the range of 10 to 100 nm is sufficient in activating ACE2 expression in macrophages. **C)** All glucocorticoids imparted a suppressive effect on IL-6 secretion. Dexamethasone revealed the strongest activating effect, followed by hydrocortisone, methylprednisolone, triamcinolone, and prednisolone. The chemical rigosertib showed a suppressive effect on IL-6 production to some extent. **D)** Low-dose hydrocortisone within the range of 50 to 100 nm is sufficient in suppressing IL-6 secretion in M1 macrophages. “*” represents a significant difference compared to the “control” or “0 nm” group. *P < 0.05, **P < 0.01, ***P < 0.001.

In view of autopsy findings by our medical rescue team in Wuhan that macrophages were aggregated in alveoli and showed elevated ACE2 and cytokine interleukin-6 (IL-6) expression, we induced human monocyte THP-1 cells into M0 (non-activated) and M1 (activated) macrophages, and then tested its activating effect of the above chemicals on ACE2 expression and its inhibitory effect on IL-6 production. After treatment of M0 or M1 macrophages with 100 nm glucocorticoids or chemicals for 48 h, we examined the protein expression levels of ACE2. Several glucocorticoids and candidate agonists revealed activating ACE2 expression in M0 macrophage, while only glucocorticoids imparted an activating effect on M1 macrophages (Figure 3A). Because hydrocortisone induced the strongest effect on activating ACE2 expression in both M0 and M1 macrophages, we further examined its effective doses using a range from 0 to 500 nm and found that a low dose of 10 to 100 nm is sufficient in activating ACE2 expression in macrophages (Figure 3B). Because IL-6 is secreted by M1 macrophages, we incubated M1 macrophages with 100 nm hydrocortisone for 48 h, and then examined IL-6 content in the supernatant. Compared to the blank control (188.8 pg/mL), all glucocorticoids showed a suppressive effect on IL-6 secretion, although the effect of dexamethasone (78.2 pg/mL) was the strongest, followed by hydrocortisone (139.9 pg/mL), methylprednisolone (157.4 pg/mL), triamcinolone (162.3 pg/mL), and prednisolone (176 pg/mL) (Figure 3C). The chemical rigosertib also imparted a suppressive effect on IL-6 production. We further examined the effective doses of hydrocortisone on suppressing IL-6 and found that low-dose hydrocortisone within the range of 50 to 100 nm is sufficient in suppressing IL-6 secretion in M1 macrophages (Figure 3D).

### Utilization of glucocorticoids as ACE2 agonist for the treatment of severe or critical COVID-19

**Figure 4:**
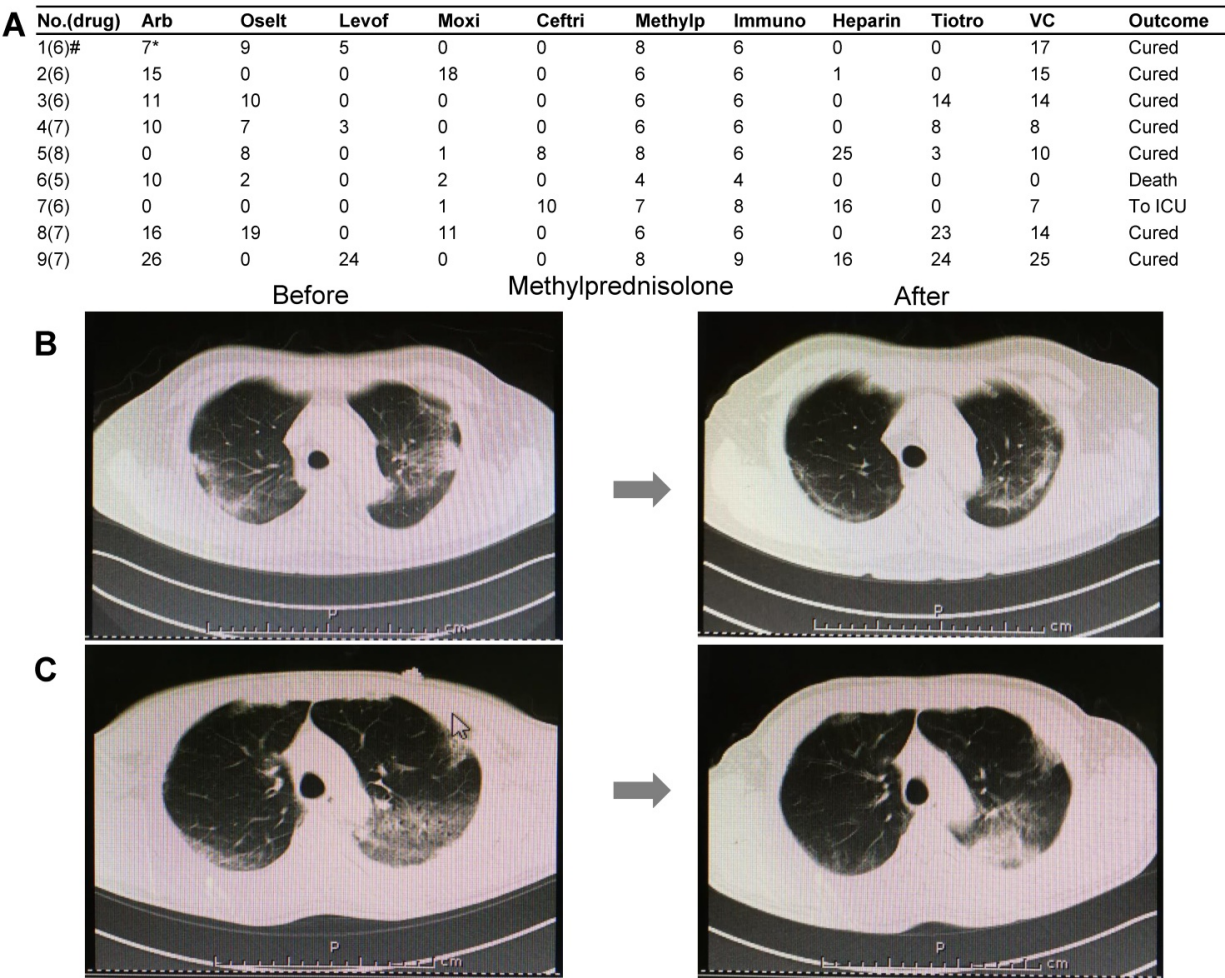
** Clinical medication details and improved CT images of nine severe or critical COVID-19 cases.** A) The drug names and treatment duration of nine cases. **B)** Pulmonary CT images before and after methylprednisolone therapy in a case of a 59-year-old man. Left: CT images were obtained before glucocorticoid treatment. Multiple lesions were found in bilateral lungs. Right: CT images obtained after 6-day treatment with glucocorticoids. Absorption of inflammatory lesions in bilateral lungs was observed. **C)** Pulmonary CT images before and after treatment by methylprednisolone in a case of a 57-year-old man. Left: CT images were obtained before glucocorticoid treatment. Multiple lesions were observed in bilateral lungs. Right: CT images obtained after 8-day methylprednisolone treatment. Inflammation absorption of bilateral lungs to some extent was observed. Drug abbreviations and dosage: Arb: Arbidol (200 mg tid PO); Oselt: Oseltamivir (75 mg bid PO); Levof: Levofloxacin (500 mg/d); Moxi: Moxifloxacin (400 mg/d); Ceftri: Ceftriaxone (2 g/d); Methylp: methylprednisolone; Immuno: Human Immunoglobulin (10 to 15 g/d); Heparin: Low-molecular weight heparin (4,000 IU/d); Tiotro: Tiotropium bromide (400 mg/d PO); VC: Vitamin C (1.5 g/d PO).

A total of 90 COVID-19 cases were treated by our medical team. Among these, nine severe or critical cases underwent glucocorticoid (methylprednisolone) treatment based on the version 7 guidelines of the National Health Commission of China. Besides oxygen therapy, antiviral therapy, and anti-infection therapy based on the situation, all nine patients were treated with medium-to-low-dose glucocorticoid (methylprednisolone). The medication information is summarized in Figure 4A. The therapeutic dose of methylprednisolone is as follows: 40 mg/d if body weight ≤ 80 kg for the first 3-4 days, and then 20 mg/d for the next 3 days or more with a total of less than 8 days. If body weight is over 80 kg, 80 mg/d for 3-4 days and 40 mg/d for the next 3 days or more with a total of less than 8 days (Table 2). We carefully observed the pulmonary CT images and peripheral lymphocyte and serum cytokine levels before or after glucocorticoid treatment. The main parameters assessed in this study included lymphocyte count and C-reactive protein (CRP) and cytokine IL-6 levels (Table 2), which significantly improved with glucocorticoid treatment. Upon radiological examination, we observed various degrees of inflammation absorption in bilateral lungs (Figures 4B and 4C) in seven out of nine cases. Two cases were terminated, which consisted of one death due to cytokine storm and another that deteriorated and was transferred to the ICU (Table 2, Cases 6 and 7).

## Discussion

ACE2 is the target of SARS-CoV-2 infection. Several reports have shown that the epithelium of the upper respiratory/digestive tract, gastrointestinal tract, or other tissues expressed higher levels of ACE2 9-11. Physiologically, ACE2 plays an important role in maintaining functional balance in vital organs, especially in the respiratory and cardiovascular systems 12,13. Imai and colleagues found that when ACE2 was knocked out in mice, these would develop acute respiratory distress syndrome (ARDS) due to S protein infection of SARS-CoV 10. The binding of viral S protein to ACE2 would deplete the host of ACE2, resulting in insufficient protection of the respiratory system, cardiovascular system, and other important organs. Moreover, patients with severe or critical COVID-19 basically receive oxygen therapy. Fang and coworkers reported that when experimental mice were exposed to a hyperoxic environment for 72 h, the lungs would exhibit hyperoxic injury accompanied by decreased ACE2 expression. They used an ACE2 agonist to increase ACE2 levels in the lung tissues and inhibited pulmonary inflammation and oxidative stress 17. Recently, our colleagues reported the results of post-mortem autopsy of two COVID-19 cases in Wuhan, China and found that numerous macrophages had aggregated in the alveoli. These macrophages expressed higher ACE2 and IL-6. The autopsy study suggested that the target cells of SARS-CoV-2 include alveolar macrophages in addition to alveolar epithelium. It has been suggested that cytokine storm is triggered by the release of IL-6 by alveolar macrophages 18. Khan and coworkers reported the results of an international multi-center clinical trial in ARDS patients with or without using recombinant human ACE2 (rhACE2). All patients who were given rhACE2 showed better tolerance and improvement, including a significant reduction in IL-6 19. Because severe or critical COVID-19 patients are subjected to ACE2 depletion, oxygen stress, and cytokine storm, it is essential to deliver ACE2 agonists during therapy.

To rapidly identify potential drugs of ACE2 agonists, we extracted differential gene sets between ACE2^high^ and ACE2^l^°^w^ cell lines from the public database and used these as inputs into the CMap database for drug repositioning. The CMap database is an extensively used database in drug repositioning research. For instance, Smalley and colleagues used this strategy and identified candidate therapeutic compounds for Huntington's disease, and confirmed that both deferoxamine and chlorzoxazone can improve neurodegeneration lesions by further experimentation 20. Drug repositioning research relies on newly developed bioinformatics. Accumulating results using human genomics and pharmacogenomics provide us valuable resources for drug screening. For example, the CCLE and CMap databases used in the present study are open databases. The former is a collection of transcriptome information before and after treating 1,019 tumor cell lines from 26 types of human tissues by 24 kinds of compounds 21. The latter is a database of transcriptome information before and after treating nine human tumor cells by 2,837 compounds 22. Based on our screening, glucocorticoids were ranked in the top 20 candidate agonists of ACE2.

Glucocorticoids are old anti-inflammatory drugs that have been shown to be very effective in treating asthma and can quickly inhibit the transcription of pro-inflammatory cytokines such as IL-2, IL-3, IL-4, IL-5, and IL-6^23^. Examples of glucocorticoids include prednisolone, triamcinolone, methylprednisolone, and dexamethasone. These drugs are all based on cortisone (hydrocortisone), which has a structural modification that enhances its anti-inflammatory effects. Based on its biological half-life *in vivo*, glucocorticoids could be further classified based on its length of effect, i.e., short-duration (8 to 12 h), medium-duration (12 to 36 h), and long-duration (36 to 72 h) drugs. For example, cortisone (hydrocortisone) has a biological half-life of 8 to 12 h. Prednisolone, methylprednisolone, or triamcinolone have a biological half-life of 12 to 36 h, whereas that of dexamethasone is 36 to72 h 24, 25. Although triamcinolone, prednisolone, and methylprednisolone are medium-duration drugs, methylprednisolone is preferred for the treatment of lung diseases because it achieves higher concentrations in the lung. Dexamethasone is preferred for the treatment of central nervous system diseases because the penetrating ability into cerebrospinal fluid is superior compared with others 26. Hydrocortisone is functionally identical to corticoids, but this name is used to distinguish it from endogenous corticoids. As an anti-inflammatory drug, hydrocortisone has been used in the treatment of septic shock at a recommended dose of 200 mg/day and via intravenous administration. Ngaosuwan and colleagues proposed that, in order to reduce steroid-associated complications such as hyperglycemia, a hydrocortisone dose of 100 mg/day might be sufficient. The patients exhibited significantly lower hyperglycemic rates compared with 200 mg/day without increasing mortality in septic shock 27.

Based on the experience during the SARS outbreak in 2002 to COVID-19 outbreak in 2019, the use of glucocorticoids in therapy has remained controversial. Shang and colleagues recently reported that low-to-moderate doses of glucocorticoids can be used for the treatment of severe or critical COVID-19, but for not more than 7 days 28. However, Russell and others expressed a different view, based on the side effects of glucocorticoids during SARS and MERS outbreaks 29. Recently, the NIH of the USA released treatment guidelines for COVID-19, which proposed that in mechanically ventilated adults with COVID-19 and ARDS, there are insufficient data to recommend either for or against corticosteroid therapy in the absence of another indication, but in COVID-19 patients with refractory shock, low-dose corticosteroid therapy is preferred over no corticosteroid therapy (https://covid19treatmentguidelines.nih.gov/). Our study provides convincing evidence that using low-to-medium-dose glucocorticoids for no more than 8 days is safe and beneficial to patients with severe-to-critical COVID-19. However, according to our experiments, hydrocortisone revealed stronger effects on activating ACE2 and inhibiting IL-6 than other glucocorticoids. The discrepancy in the results of our experimental study and bioinformatics prediction may be attributable to differences in cell origins. In bioinformatics prediction, tumor cell lines databases were used, whereas in validating our experiments, immortalized normal cell lines were used. Therefore, the results of any *in silico* study should be verified by laboratory experiments. Because hydrocortisone resulted in better protective effects on both epithelial cells and macrophages, moderate- to low-dose hydrocortisone (200-100 mg/day) may be appropriate in treating severe or critical COVID-19 relative to methylprednisolone. However, this needs to be verified in the clinic. In addition, other candidate ACE2 agonists such as kinetin riboside, cilomilast (Ariflo, SB-207499), and rigosertib showed activating effects on ACE2 expression or inhibiting effects on IL-6 production to some extent. However, these were not stronger than glucocorticoids. Whether these could be used in COVID-19 treatment thus requires further investigation.

In conclusion, with the current settings in drug research, the development of new drugs within a short period of time remains a major challenge. Drug repositioning provides a new approach in identifying drugs for specific diseases. The results of this study may be used as a resource in the design of COVID-19 therapeutic agents.

## Materials and Methods

### Extraction and analysis of transcriptomic data

Transcriptomic data of 1,019 tumor cell line from 26 kinds of human tissues was downloaded from the CCLE database (https://portals.broadinstitute.org/ccle). The mRNA expression level of ACE2 was analyzed. The data were divided into three groups based on the expression level of the ACE2 gene, i.e., high expression group (n=340), medium expression group (n=339), and low expression group (n=340). Then, we further compared differential gene sets between the ACE2^high^ and ACE2^l^°^w^ groups and obtained two gene sets from each group. The top 150 genes with the most significant differences in above two gene sets (fold-change > 1.5-fold, P < 0.01) were used as input into the Connectivity Map database (CMap, https://clue.io/), and matched corresponding compounds that could cause the above gene profiling changes. The resulting compounds with positive scores were designated as candidate agonists. The full-mark score is 100. When the score is closer to 100, the candidate compound is more credible.

### Cell lines

Human immortalized gastric epithelium GES-1 and human monocyte THP-1 were cultured in RPMI-1640 containing 10% FBS and 1% penicillin-streptomycin and incubated at 37°C in a humidified incubator at 5% CO_2_. THP-1 cells were induced in RPMI-1640 with 100 ng/mL phorbol 12-myristate 13-acetate (PMA) for 24 h for macrophage (M0), and then was further incubated for another 24 h with 20 ng/mL IFN-γ and 10 ng/mL lipopolysaccharide (LPS) for the activation of macrophages (M1).

### Reagents

Glucocorticoids methylprednisolone and triamcinolone were purchased from MCE (New Jersey, USA). Dexamethasone, hydrocortisone, and prednisolone were purchased from SELLECK (Houston, TX, USA). Chemical triciribine was obtained from SELLECK. Kinetin riboside, cilomilast, rigosertib, PMA, and LPS were purchased from MCE Co. IFN-γ was from PeproTech (New Jersey, USA).

### Protein expression analysis

The cell lines were incubated with different chemicals for 48 h, followed by total protein extraction. The protein expression levels of ACE2 (rabbit anti-human ACE2, 1:1,000, AB15348, Cambridge, UK) and HRP-conjugated GAPDH (1:3000, Proteintech, Wuhan, China) were detected by western blotting.

### Human IL-6 detection

The M0 and M1 macrophages were incubated in the above drugs for 48 h, then IL-6 in the supernatants was detected by ELISA. A human IL-6 test kit was purchased from Novus (Colorado, USA). Detection was performed according to the product's instructions.

### COVID-19 patients

A total of 90 COVID-19 patients were admitted to our medical team. Nine of these cases were evaluated as severe or critical COVID-19 and received glucocorticoid treatment according to the version 7 guidelines of the National Health Commission of China. Severe COVID-19 was diagnosed using the following criteria: (1) respiratory distress (≥ 30 breaths/min); (2) oxygen saturation ≤ 93% at rest; (3) arterial partial pressure of oxygen (PaO_2_)/fraction of inspired oxygen (FiO_2_) ≤ 300 mmHg (l mmHg = 0.133kPa). Cases with chest imaging that showed obvious lesion progression within 24-48 h of > 50% were managed as severe cases. Patients meeting any of following conditions were diagnosed as critical COVID-19: (1) respiratory failure and requiring mechanical ventilation; (2) shock; (3) other organ failure that requires ICU care 30. To evaluate the therapeutic effects, the parameters of lymphocyte ratio and C-reactive protein and IL-6 cytokine levels were determined before and after glucocorticoid therapy. Chest CT scans of each patient were examined. The clinical study was approved by the Ethics Committee of Ruijin Hospital in Shanghai.

### Statistics

All results of IL-6 detection were expressed as the mean ± SD of three repeats. The Student's t-test was conducted using GraphPad Prism 6.0 (CA, USA). Differential gene profiles were plotted using hierarchical clustering by “corrplot” package in R software. Differences with P values < 0.05 were considered significant.

## Figures and Tables

**Table 1:** The top 20 candidate ACE2 agonists

Name	Function	CMap score
Cytochalasin-d	Actin polymerization inhibitor, actin stabilizer	98.4
Flubendazole	Microtubule inhibitor, anthelmintic drug	98.2
Triciribine*	DNA synthesis inhibitor, AKT inhibitor, HIV inhibitor	97.8
Rigosertib*	Multi-kinase inhibitor, cell cycle inhibitor, apoptosis inducer	97.7
Prostratin	NFkB activator, protein kinase activator, HIV inhibitor	95.2
HG-5-88-01	Protein kinase inhibitor	94.7
Quinoclamine	Algicide	94.4
HU-211	Glutamate receptor antagonist, neuroprotective, antioxidant	93.9
CS-1657	PARP inhibitor	93.6
Deforolimus	mTOR inhibitor, angiogenesis inhibitor, immunosuppressant	93.1
Kinetin-riboside*	Apoptosis inducer, anti-proliferative agent	91.8
Vinorelbine	Mitosis inhibitor, microtubule inhibitor, apoptosis inducer	91.6
Triamcinolone*	Corticosteroid hormone receptor agonist, anti-inflammatory agent	91.3
Perospirone	Dopamine receptor antagonist, antipsychotic agent	91.2
OMDM-2	Anandamide uptake inhibitor	91.1
Eicosatetraynoic-acid	Cyclooxygenase inhibitor, Arachidonic acid uptake inhibitor	91.0
Cilomilast*	Phosphodiesterase inhibitor, for respiratory disorders (COPD)	90.9
BRD-K28680267	Cholecystokinin (CCK) receptor inhibitor	90.7
Desoxycortone	Mineralocorticoid receptor agonist	90.7
Phensuximide	Anticonvulsant	89.1

*Represents the available compounds for subsequent validation experiments

**Table 2:** Therapeutic details of nine severe-to-critical COVID-19 cases using medium-to-low-dose methylprednisolone

Case	Age /Sex	Confirmed date$	Oxygen therapy#	Oxygen intake#	Disease type	Vital signs at admission				Lymphocyte Before/after*	CRP Before/after*	IL-6 Before/after*	Methylpred treatment time	CT Scan Before/after*	Outcome
						HR	BP	SaO_2_	RR						
1	79/F	7-day	Nasal catheter/ oxygen mask	3 L/min	Critical	115	104/77	86	35	0.58/0.67	93.7/5.4	35.33/5.54	8-day	Yes/Yes	Cured
2	76/F	7-day	Nasal catheter/ oxygen mask	10 L/min	Critical	61	112/54	97	20	0.43/0.48	118.4/14.7	5.0/5.1	6-day	Yes/Yes	Cured
3	45/F	13-day	Nasal catheter	5 L/min	Severe	88	128/81	95	20	1.20/1.24	5/0.5	ND/ND&	6-day	Yes/Yes	Cured
4	32/F	14-day	Nasal catheter	5 L/min	Severe	78	107/68	97	30	2.63/2.09	2.6/0.5	ND/ND&	6-day	Yes/Yes	Cured
5	44/M	2-day	Nasal catheter/ intubation, ECMO	6 L/min	Severe	78	169/90	95	18	0.71/1.28	19.5/ND	16.98/3.01	8-day	Yes/Yes	Cured
6	69/F	12-day	Oxygen mask/ intubation	6 L/min	Critical	84	195/101	93	14	0.58/ND	62.4/ND	24.26/ND	4-day	Yes/No	Death
7	68/F	22-day	Nasal catheter/ intubation	6 L/min	Critical	91	130/85	84	32	0.98/1.00	62.7/>320	17.48/169	7-day	Yes/No	To ICU
8	59/M	11-day	Nasal catheter	3 L/min	Critical	122	132/87	96	18	0.75/1.83	119.1/8.2	ND/ND&	6-day	Yes/Yes	Cured
9	57/M	16-day	Nasal catheter	3 L/min	Severe	124	141/84	91	28	1.06/1.52	68.1/4.4	56.4/9.94	8-day	Yes/Yes	Cured

Note: $ from appearing symptom to positivity for viral nucleic acid detection. # represents the status of giving oxygen. *represents count or level before or after methylprednisolone therapy. ND: not done. ND& means that IL-6 levels were not assessed during the early outbreak stage in the Wuhan hospital. Methylpred: methylprednisolone. SaO_2_: arterial oxygen saturation.
